# Molecular Dynamics Simulations of Insulin: Elucidating the Conformational Changes that Enable Its Binding

**DOI:** 10.1371/journal.pone.0144058

**Published:** 2015-12-02

**Authors:** Anastasios Papaioannou, Serdar Kuyucak, Zdenka Kuncic

**Affiliations:** 1 Charles Perkins Centre, University of Sydney, Sydney, NSW, Australia; 2 School of Physics, University of Sydney, Sydney, NSW, Australia; University of Technology Sydney, AUSTRALIA

## Abstract

A sequence of complex conformational changes is required for insulin to bind to the insulin receptor. Recent experimental evidence points to the B chain C-terminal (BC-CT) as the location of these changes in insulin. Here, we present molecular dynamics simulations of insulin that reveal new insights into the structural changes occurring in the BC-CT. We find three key results: 1) The opening of the BC-CT is inherently stochastic and progresses through an open and then a “wide-open” conformation—the wide-open conformation is essential for receptor binding, but occurs only rarely. 2) The BC-CT opens with a zipper-like mechanism, with a hinge at the Phe24 residue, and is maintained in the dominant closed/inactive state by hydrophobic interactions of the neighboring Tyr26, the critical residue where opening of the BC-CT (activation of insulin) is initiated. 3) The mutation Y26N is a potential candidate as a therapeutic insulin analogue. Overall, our results suggest that the binding of insulin to its receptor is a highly dynamic and stochastic process, where initial docking occurs in an open conformation and full binding is facilitated through interactions of insulin receptor residues with insulin in its wide-open conformation.

## Introduction

**Fig 1:**
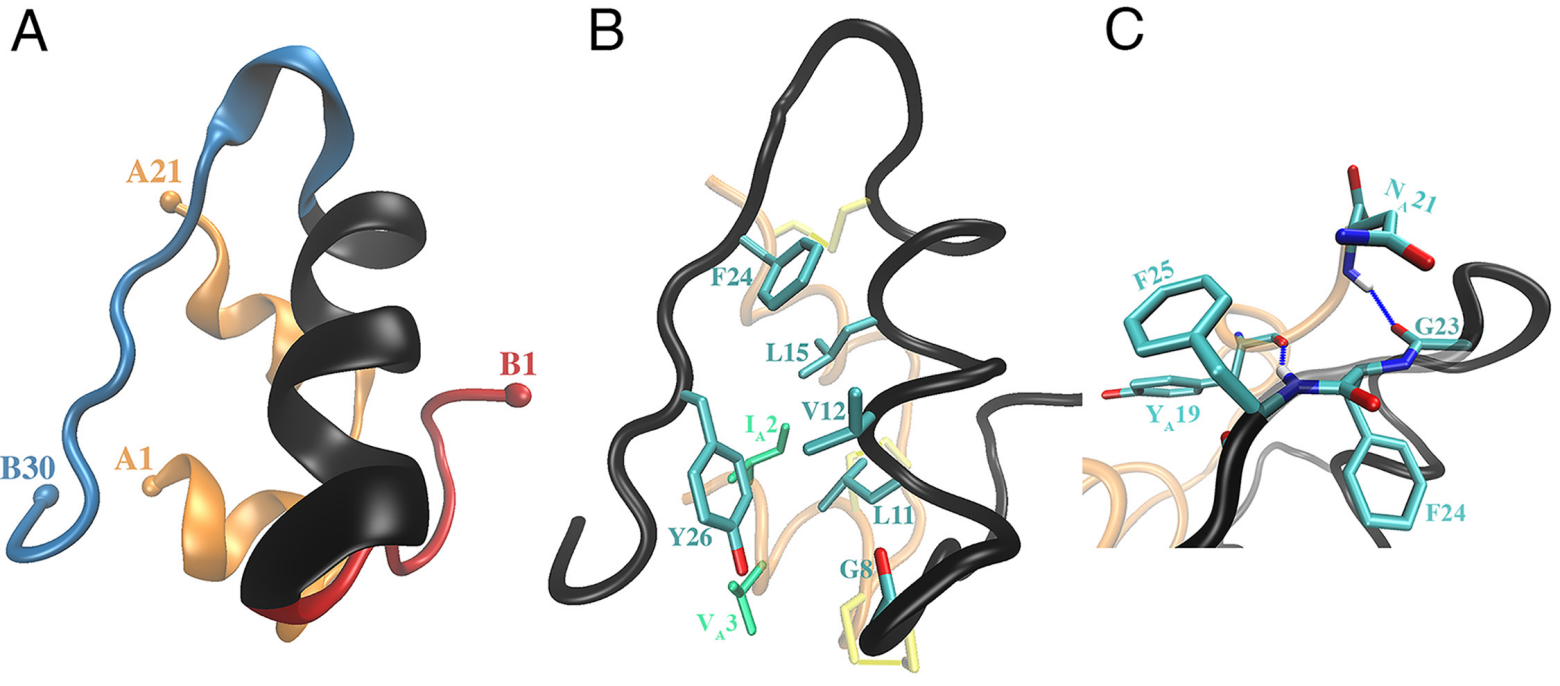
Structural details of the insulin molecule. **(A)** The structure of the insulin (PDB ID: 2G4M). A-Chain (orange); B-chain N-terminal (residues F1–G8) (red), B-chain α-helix (residues S9–C19) (black), B-chain C-terminal (residues G20–T30) (blue). The colored spheres represent the C_α_ atoms of the first and last residues of the A and B-chains. **(B)** The new ribbon representation shows the hydrophobic core of insulin, comprising the residues F24, L15, Y26, L11, V12, G8 (side chains shown in cyan), I2_A_ and V3_A_ (side chains shown in green). The hydroxyl group of Y26 and the oxygen atom of G8 are shown in red, and the disulfide bridges in yellow. **(C)** The hydrogen bonds between the residues neighboring Y24 and the A chain residues, G23(O)–N2_A_(N) and F25(N)–Y19_A_(O), are illustrated in blue dashed lines. Oxygen and nitrogen atoms are represented in red and blue, respectively.

Insulin is a polypeptide hormone that plays a crucial role in regulating glucose levels in higher organisms. Defects in insulin signalling can lead to insulin resistance, which is the hallmark of diabetes mellitus. The key molecular mechanisms and kinetics in the insulin-signaling pathway have been extensively studied but a complete picture is yet to emerge. In particular, the dynamics of how insulin binds to the insulin receptor (IR) and activates signal transduction needs to be better understood for pharmacological applications. Insulin comprises two polypeptide chains, a 21 amino acid A-chain made of two α-helices, and a 30 amino acid B-chain containing a central α-helix (see Fig 1*A*). The two chains are constrained by two inter-chain (A7–B7 and A20–B19) and one intra-chain (A6–A11) disulphide bridges (see Fig 1*B*). The first crystal structure of insulin was determined by Adams et al. in 1969 [1] followed by other structures of insulin and its analogs in the storage form (hexamers and dimers) [2,3]. Insulin monomer triggers signal transduction through binding to the tyrosine kinase-type IR with high affinity, which is a disulphide-linked (αβ)_2_ homodimer [3]. Two surfaces on the insulin molecule have been identified to interact with the IR [4]. The first comprises predominantly the hormone-dimerizing residues and interacts with the αCT segment from one IR α-chain and the central β-sheet of the L1 domain of the other α-chain (primary binding site) [4–9]. The second comprises predominantly the hormone-hexamerizing residues and is suggested to interact with the IR site at the junction of FnIII-1 and 2 of the α-chain (site 2) [4,6–11].

Attempts to dock insulin to the IR using the available crystal structures have been unsuccessful to date [12,13], indicating that conformational changes are required to enable its binding. Several recent experimental studies [14–20] have further revealed that substantial conformational changes occur in both insulin and the primary site of IR during binding. These are: initial detachment (opening) of the B-chain C-terminal (BC-CT) of insulin from its hydrophobic (helical) core to expose key binding residues (see Fig 1*A* and 1*B*), followed by a rotation and re-modeling of the αCT portion of IR upon the surface of the β-sheet of the L1 domain of IR, and lastly docking of the insulin B-chain α-helix in a parallel orientation to the αCT segment, creating strong hydrophobic interactions in between. Extensive mutagenesis and structural studies of insulin substantiate the critical role of the BC-CT, and in particular the residues F24–Y26, in binding to IR [17,18,21–25]. Furthermore, the first crystal structure of the insulin–IR complex has revealed the extent of the structural changes and the importance of the BC-CT residues of insulin, especially F24-Y26, as well as the crucial role of H710 and F714 of the αCT segment [14,26]. In a recent experimental study [15], a hinge in the BC-CT (F24 residue) was identified, which is critical for insulin to engage the αCT segment of IR (see Fig 1*B* and 1*C*). In contrast to these numerous experimental studies of the insulin-IR complex, there have been relatively few computational studies investigating the conformational and dynamic properties of insulin [17,24,27,28]. Furthermore, all the previous experimental and computational studies on insulin focused on the structural changes that occur in the BC-CT during activation and on mutations in the critical residues, F24 (all the residues mentioned without extra labelling belong to chain B. The A chain residues are denoted by a subscript A) and Y26 [15,17–19,21–25,27,28]. Two key questions regarding the activation of insulin remain unanswered: the frequency of the BC-CT opening, which is necessary for insulin to bind to the IR, and the mechanism that triggers this opening. We, therefore, performed MD simulations of wild-type (WT) and mutated insulin to gain new insights into the active form of insulin, i.e., the opening of the BC-CT, and the behavior of its hydrophobic core consisting of the residues G8, L11, V12, L15, F24 and Y26 from the B-chain and I2_A_ and V3_A_ from the A-chain (see Fig 1*B*). The opening of the BC-CT was studied by examining the distances between the neighboring BC-CT and α-helix residues (see Table 1), and constructing the potential of mean force (PMF) from the distance distributions. Finally, the mechanism that triggers opening of the hydrophobic core was studied via mutations of the critical residues on the BC-CT and observing the behavior of water molecules in the immediate vicinity of the core.

**Table 1:** The BC-CT and B-chain α-helix residues used to characterize the dynamics of the BC-CT.

C_α_ atom of the BC-CT residue and the closest C_α_ atom in the B-chain α-helix	Distances in the crystal structure 2G4M [Å]	Average distances from MD simulations where insulin is in the closed state [Å]
F24(C_α_)–L15(C_α_)	6.7	7.0±0.4
F25(C_α_)–L15(C_α_)	8.5	9.4±0.5
Y26(C_α_)–V12(C_α_)	7.2	8.1±0.9
T27(C_α_)–V12(C_α_)	10.2	10.5±1.1
P28(C_α_)–G8(C_α_)	9.1	9.8±2.1
L29(C_α_)–G8(C_α_)	12.2	12.3±2.4
T30(C_α_)–G8(C_α_)	14.5	13.8±2.7

## Methods

### Insulin Modelling

In the MD simulations, we used a crystal structure of insulin with a resolution of 1.8 Å (PDB ID: 2G4M [29]). All simulations were constructed using the VMD software following the same protocol. The structure of insulin was solvated with approximately 3500 water molecules. Water molecules associated with the crystal structure, which help to stabilize the protein, were also included in the simulation box. Nine Na^+^ and seven Cl^-^ ions were placed randomly in the solvated system to neutralize it and to set the NaCl concentration to 0.1 M. The extra 2 Na^+^ ions are needed to neutralize the negative charges of the protein and thus avoid any artifacts arising from the use of periodic boundaries. The simulations were then subjected to an initial minimization of water molecules followed by equilibration. The *x*, *y* and *z* dimensions of the water box were approximately 54, 52 and 45 Å, respectively. In the first stage of equilibration, the system was equilibrated with 1 atm pressure coupling until correct water densities were obtained. In the next stage, the restraints on the side chain and backbone atoms of the protein were gradually relaxed by reducing them from k = 30 to 0.1 kcal/mol/Å^2^. Finally, the protein was released, i.e., all restraints on the protein were removed, and the resulting system was then ready for MD simulations. Equilibration of the system was monitored from the RMSD data.

### MD Simulations

The MD simulations were performed using the NAMD package [30] employing the CHARMM36 force field [31,32]. The NpT ensemble was used with periodic boundary conditions in combination with the Particle Mesh Ewald (PME) method, which calculates the long-range electrostatic interactions in the periodic system. The temperature and pressure were maintained at 300 K and 1 atm, respectively, by applying the Langevin coupling with a damping coefficient of 5 ps^-1^. The simulations were carried out with a switching cut-off within a distance of 10–13 Å for Lennard-Jones interactions. The list of non-bonded interactions was truncated at 13.5 Å applying an update frequency of 1 ps. The time step employed in all simulations was 2 fs and trajectory data were recorded every 10 ps over the course of the simulation.

### WT Insulin

**Fig 2:**
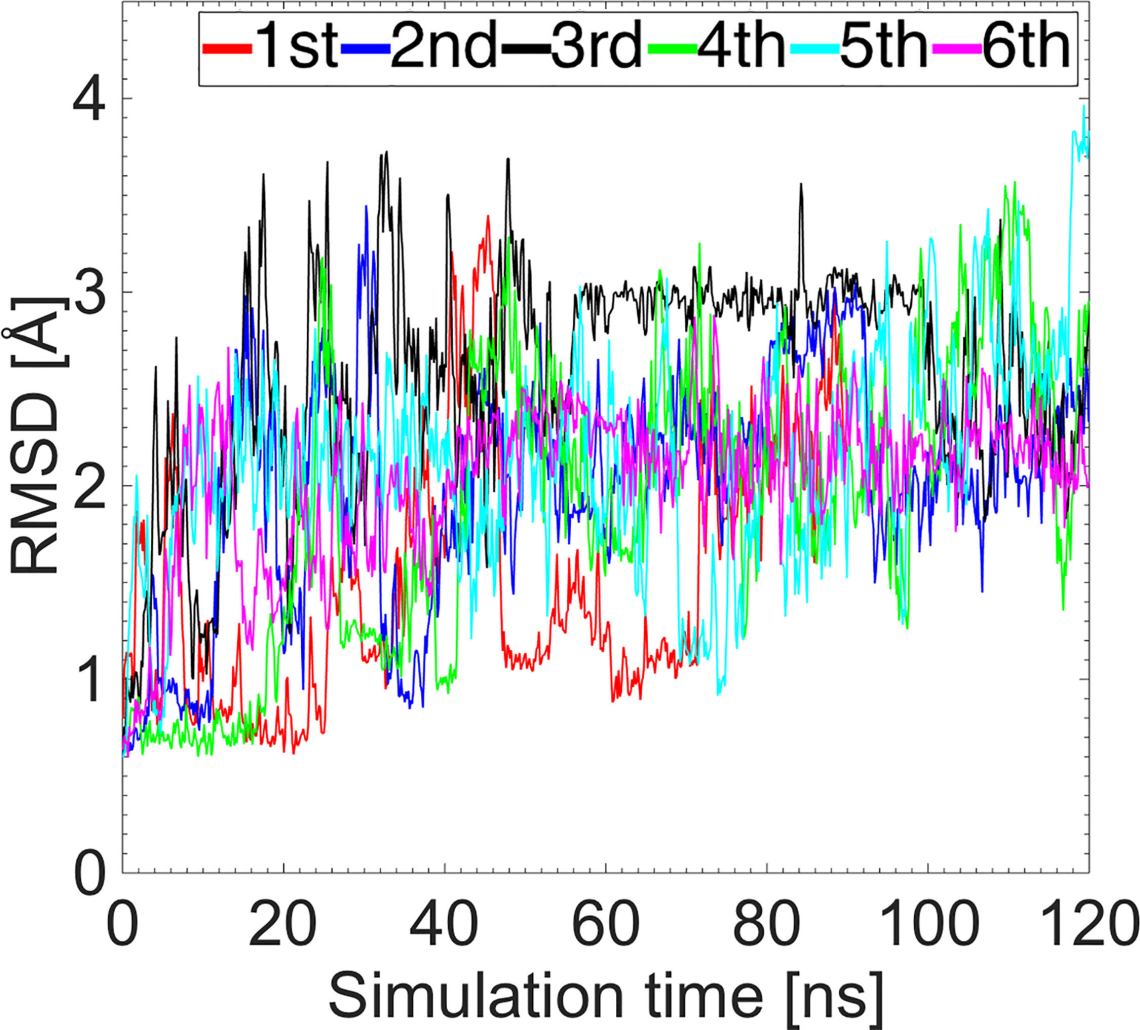
RMSDs of six MD simulations of insulin. The first five MD simulations have a duration of 120 ns, while the sixth one 90 ns. The RMSDs are given with respect to the crystal structure 2G4M.

We performed a 690 ns MD simulation for WT insulin, which was split into six shorter ones to improve sampling (5×120 ns and 1×90 ns). Different relaxation times were used for the starting configuration in each MD simulation, i.e., 250, 300, …, 500 ps. The Root Mean Square Deviations (RMSDs) of insulin for individual MD simulations are shown in Fig 2. Each RMSD has a distinct initial behavior, indicating the independence of the starting configurations. All the statistical analyses, discussed in the Results subsection “Characterization of the active form of insulin”, are based on the long MD simulation of WT insulin.

### Water molecules within the hydrophobic core

We investigated the role of water molecules in the opening of the BC-CT by calculating the number of water molecules within a sphere that covers the whole volume of the hydrophobic core during the long MD simulations. The sphere was centred on the C_γ_ atom of the L15 residue, located in the centre of the hydrophobic core, and had a radius of 9 Å. While the hydrophobic core had a few residual water molecules in the closed state, the sphere included additional water molecules outside the core, which averaged to nine for closed conformations. Thus, nine water molecules were subtracted from the total number of water molecules found in the sphere.

### Potential of Mean Force (PMF) calculation

The PMF describes how a system’s free energy changes as a function of a specific reaction coordinate. To characterize the dynamics of the BC-CT opening, we chose for this purpose the distance of the C_α_ atoms of the BC-CT residues from the closest C_α_ atoms in the B-chain α-helix (see Table 1). We note that these coordinates do not necessarily represent any direct coupling between the pairs of residues, but were chosen because they provide the best description for the opening of the BC-CT. As we had a sufficiently long sampling of insulin in the MD simulations, the PMF was determined directly from the density data using the Boltzmann equation

W=−kTln(ρρ0) (1)

where *T* = 300 K is the system temperature, *ρ* is the density obtained from distance distributions, and *ρ*_*0*_ is a reference density. With this information, we can determine how much free energy is required to change insulin’s conformation from the closed/inactive to the open/active forms.

### Mutated Insulin

We also considered several mutations on the Y26 residue to examine the behavior of the active conformation of insulin and to compare the simulation results with the experimental findings. The mutant insulin structures were created using the mutator plugin in the VMD software [33] and a 100 ns MD simulation was performed in each case using the same simulation protocol as described in the “MD simulations”.

### Breaking of the F24 hinge

In order to break the hinge at the F24 residue and facilitate a wider opening of the BC-CT, we applied a repulsive harmonic force to the C_α_ atoms of the F24 and P28 residues relative to their partners in the B-chain α-helix (see Table 1). The potential *U* used is given by

$$U=\left\{ \begin{array}{l} \frac{1}{2}k{\left(r-r_{0}\right)}^{2},\;r<r_{0}\\ 0\;,\;r>r_{0} \end{array}\right.$$ (2)

where *k* is the spring constant, *r* is the distance between the C_α_ atoms of the chosen pairs, and *r*_0_ is the target distance. A force constant of 30 kcal/mol/Å^2^ was used and *r*_0_ was determined by adding 9.8 Å to the average distances given in Table 1. This potential was applied to the C_α_ atoms of the pairs of residues F24–L15 and P28–G8 for 200 ps, which was sufficient to break the hinge. After that, a 30 ns MD simulation was performed without any forces applied to insulin.

## Results and Discussion

### Characterization of the active form of insulin

Visual inspection of the long MD simulations of WT insulin shows that it stays predominantly in the closed conformation and the BC-CT makes only occasional excursions away from the B-chain α-helix. The MD simulations of insulin in the closed state were validated by comparing with the corresponding crystal structure 2G4M. As shown in Table 1, the average distances between the C_α_ atoms of BC-CT and B-chain α-helix agree with the experimental values within 1 Å. We note that the fluctuations in distances increase from F24 to T30, which points to an effective interaction that diminishes with the distance from the hinge towards the C-terminal. As discussed in Fig 1B, the side chains of F24 and Y26 contribute to the formation of the hydrophobic core, which is responsible for maintaining insulin in the closed state. Table 2 lists the strongest hydrophobic interactions involving the F24 and Y26 side chains and compares the average distances obtained from the MD simulations with the experimental values. Again there is a good agreement between the simulation results and experiments, which further validates the MD simulations.

**Table 2:** The strong interactions in the hydrophobic core that are responsible for maintaining insulin in its closed conformation.

Interaction	Distance in the crystal structure [Å]	Average distance during MD simulations where insulin stays at its closed conformation [Å]
F24(Aromatic)–L15(C)	5.2	4.6±0.5
Y26 (Aromatic)–I2_A_(C)	5.2	5.5±2.1
Y26(Aromatic)–L11(C)	5.2	5.8±1.3
Y26(Aromatic)–V12(C)	3.8	4.5±1.0

The distances were calculated from the center of the benzene ring (aromatic) to the nearest C atom. The same trajectory data as in Table 1 were used in calculating the average distances.

**Fig 3:**
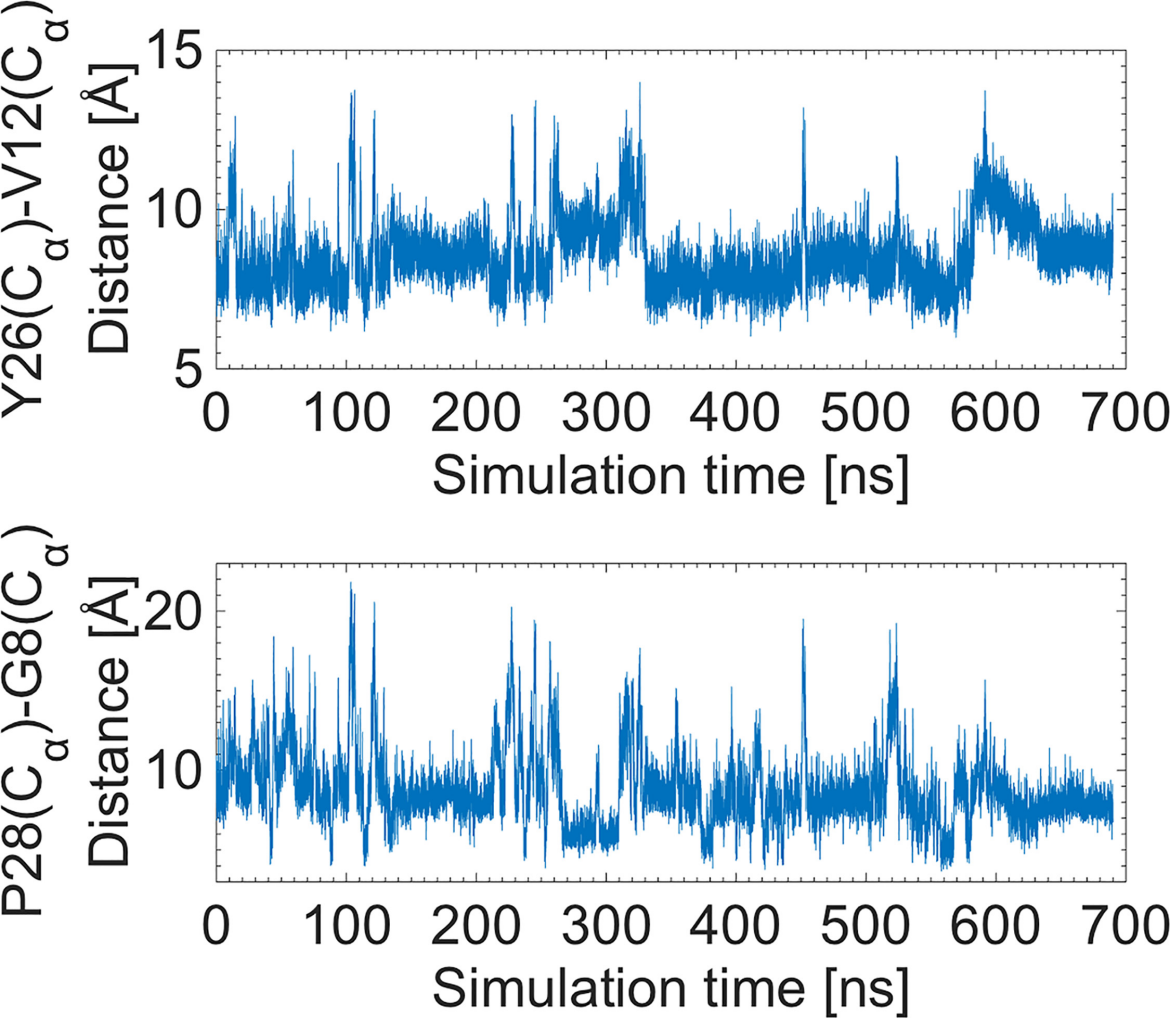
Time series of the distances between the C_α_ atoms of Y26–V12 and P28–G8. These distances are used as a criterion for the opening of the BC-CT and activation of insulin.

Examination of the time series of the distances listed in Table 1 shows that the F24–L15 distance does not change, consistent with a hinge behavior, while others increase to varying degrees. As an example, Fig 3 shows the distances between the C_α_ atoms of the pairs Y26–V12 and P28–G8 from the MD simulation. These two distances provide the most useful indicators for opening of the BC-CT, and hence warrant further consideration. Their average distances are approximately 8.1 and 9.8 Å, respectively. It is evident from all the MD simulations that there is no consistent or systematic dynamic behavior characterizing the opening like a switch or quasi-periodic motion between two states. Insulin activation instead occurs stochastically at uncorrelated simulation times and frequencies, and appears to be a random event.

**Fig 4:**
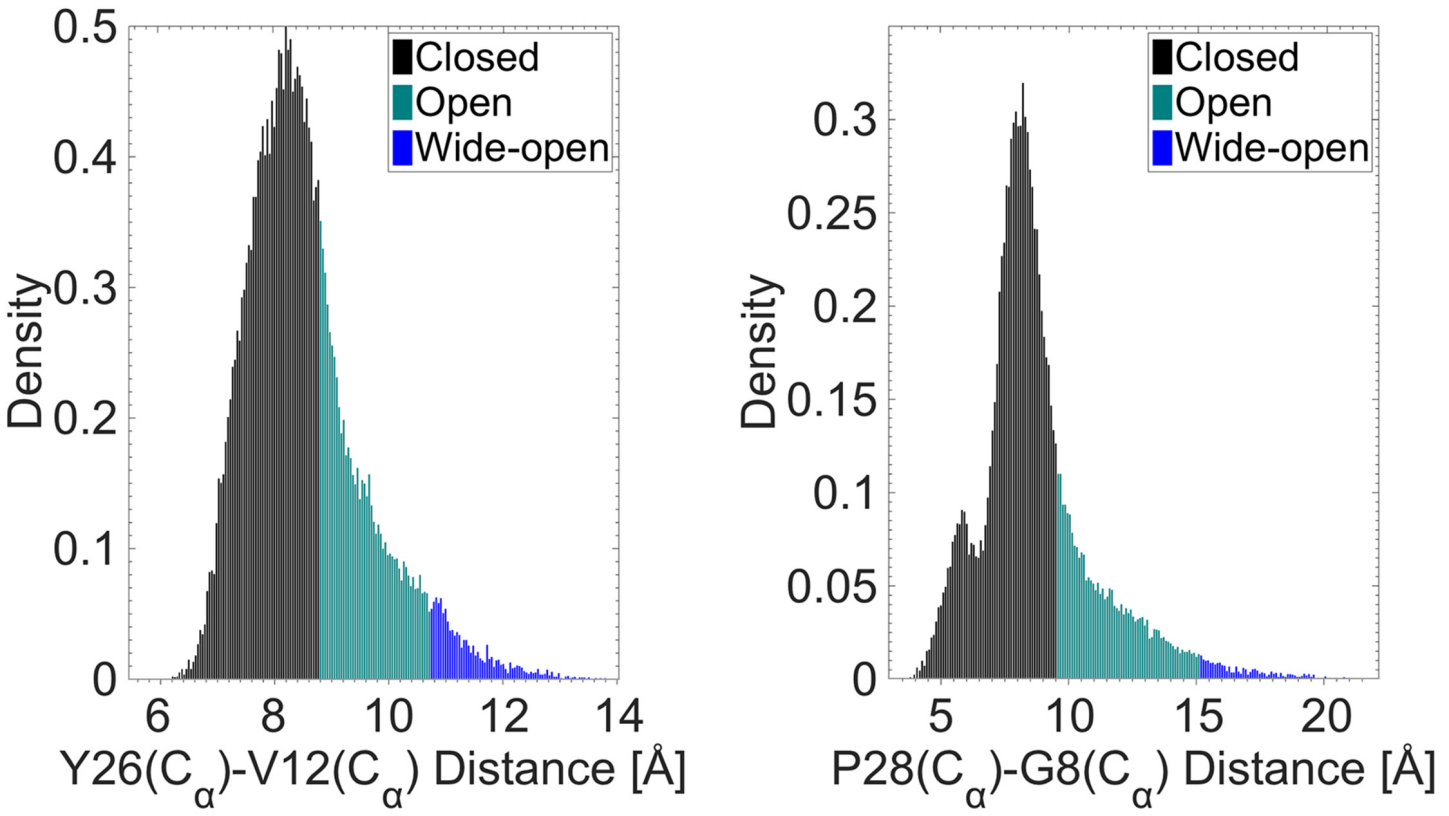
Distribution of the distances between the C_α_ atoms of Y26–V12 and P28–G8. The different histogram colors correspond to the different conformations of the BC-CT, namely, closed (black), open (cyan) and wide-open (blue).

To analyze the frequency of the opening quantitatively, Fig 4 plot the distance histograms obtained from the 690 ns of trajectory data as probability densities, representing the probability of a distance occurring during the MD simulations. The peak values in the distance distributions occur at approximately 8.2 Å for both Y26–V12 and P28–G8. The threshold for the open conformation was identified by the entry of water molecules into the hydrophobic core. A non zero number of water molecules inside the hydrophobic core indicates a transition from the closed to the open conformation. Based on this criterion, we found that a distance of less than 8.7 and 9.5 Å for the Y26–V12 and P28–G8 distributions, respectively, corresponds to the closed conformation of insulin. This is indicated by the black histograms in Fig 4, which clearly also indicates that the most preferable conformation of insulin is the closed/inactive form (64% from the Y26–V12 distribution). The probability of the open conformation (cyan histograms) decreases rapidly with increasing C_α_ atom distance. Fig 4 also suggests the existence of a third conformation, corresponding to the tail end of the distribution (blue histograms). We refer to this as the “wide-open” conformation, defined as the open conformation of insulin that enables it to fit to the IR, attributable to a ≈50° rotation (wide opening) of the F24-T30 BC-CT part about F24 [15]. The boundary between the open and wide-open conformations is found to occur when the Y26-V12 and P28-G8 distances are approximately 10.7 and 15 Å, respectively. From the distance histograms in Fig 4, the wide-open conformation has a low probability of occurrence (5% from the Y26–V12 distribution) and thus it is a relatively rare event. This suggests that initial docking of insulin to the IR may occur in an “open” conformation and the “wide-open” conformation required for full docking may be facilitated through interactions with the IR residues.

**Fig 5:**
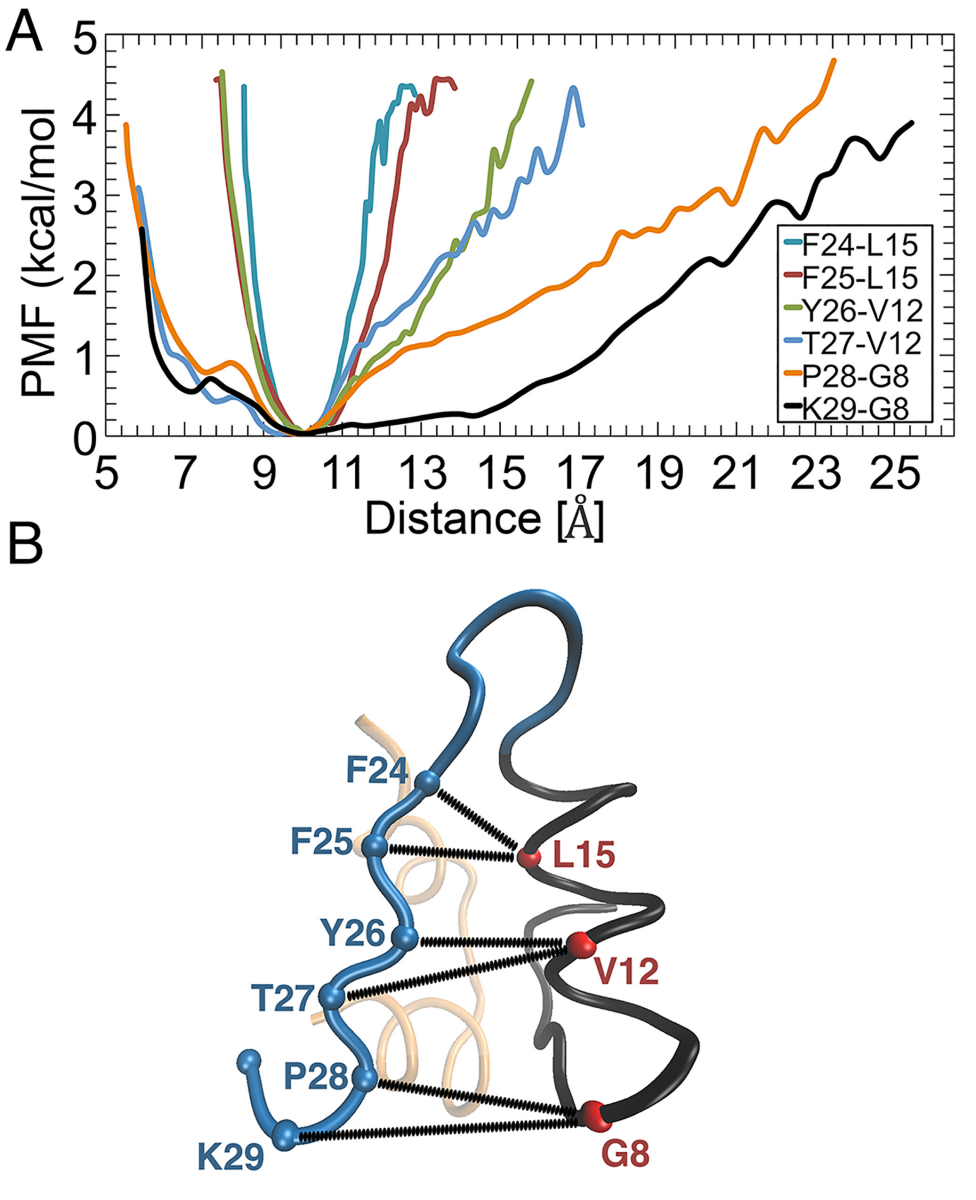
PMFs calculation. (A) The PMFs as a function of the distance between the pairs shown in Table 1. (B) Illustration of the pairs. The spheres in blue and red represent the C_α_ atoms of the BC-CT and B-chain α-helix residues, respectively.

**Fig 6:**
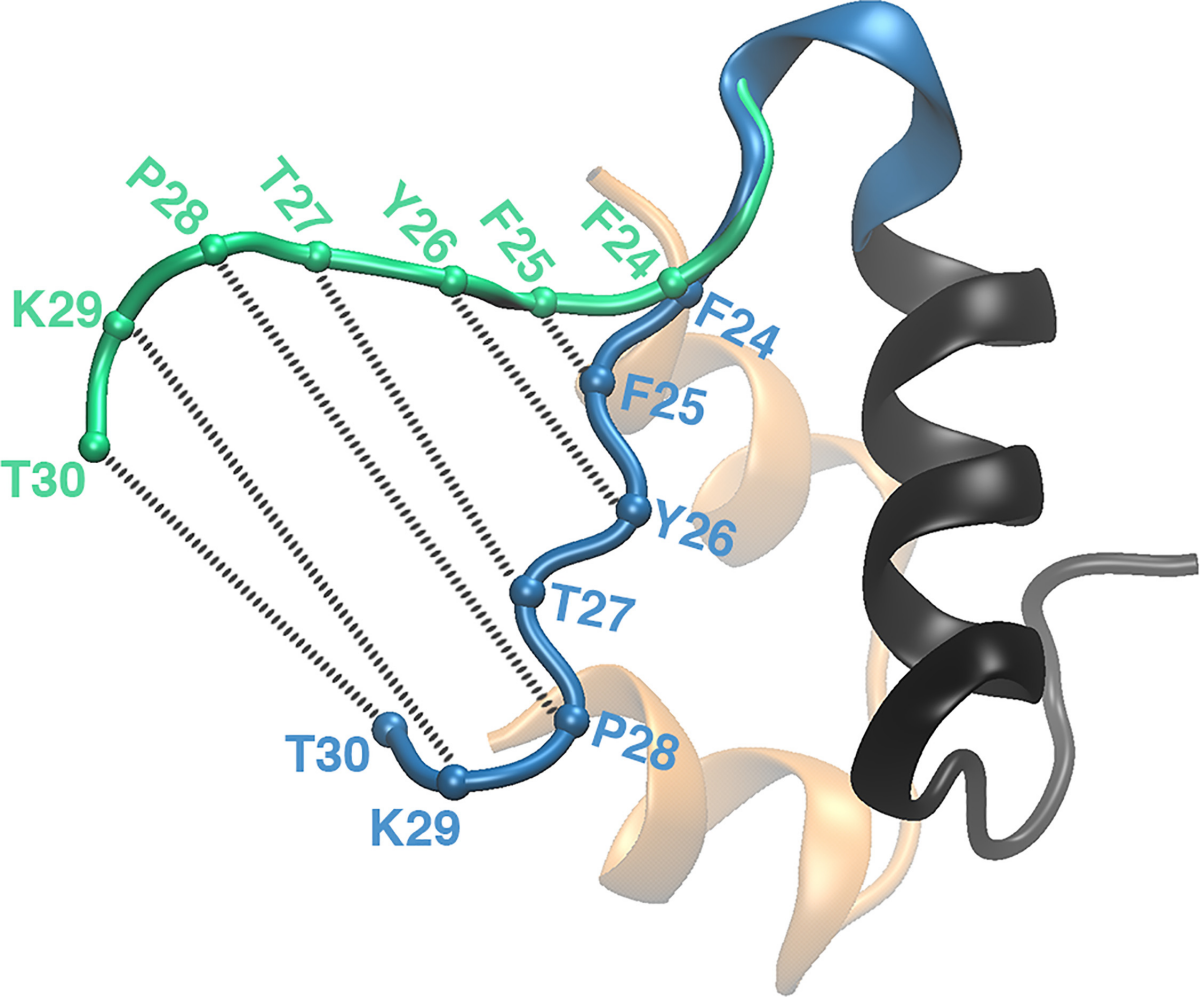
The zipper-like opening of the BC-CT with a hinge at F24. The BC-CT in its closed and open conformations is shown in blue and green, respectively. The colored spheres represent the C_α_ atoms of the corresponding residues.

We next consider the energetics of insulin opening by converting the distance distributions for the pairs listed in Table 1 to PMFs using Eq 2. Fig 5*A* shows the PMFs for all the pairs involved in the opening of insulin, plotted as a function of distances between the C_α_ atoms of the BC-CT and B-chain α-helix (cf. Table 1 and Fig 5*B*). To facilitate comparison of the PMFs, the minima are set to zero and the curves are aligned at the same equilibrium distance (because the PMFs are effective interactions, the equilibrium distances are relative, not absolute). The C-terminal residue T30 is not included in the PMFs as it moves approximately randomly. The next BC-CT residue (L29) is seen to be the most flexible. Its conformation changes from closed to open within 1 kcal/mol. The amount of energy needed for conformational changes increases systematically from L29 to the hinge residue F24. This suggests that the BC-CT opens in a zipper-like fashion. This opening is initiated in the end residues of the C-terminal and continues through successive residues on the BC-CT until the hinge residue F24 (see Fig 6).

**Fig 7:**
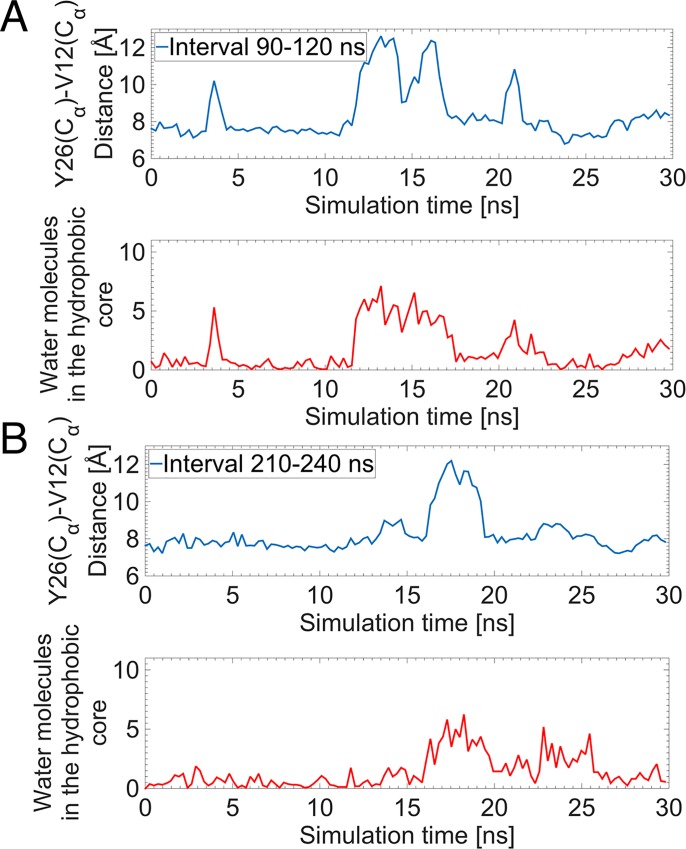
Entry of water molecules as a criterion of the BC-CT opening. Correlation between the number of water molecules within the hydrophobic core (red) and the opening of the BC-CT, i.e. the Y26(C_α_)-V12(C_α_) distance (blue), for two randomly chosen intervals of the long MD simulation. (A) 90–120 ns and (B) 210–240 ns.

Our simulations further suggest that the mechanism of insulin opening is driven by the entry of water molecules into the hydrophobic core. Fig 7 shows the correlations between the number of water molecules within the hydrophobic core with the opening of the BC-CT during two randomly selected intervals from the 690 ns MD simulations. These correlations indicate that the main criterion for the opening is the Y26(C_α_)–V12(C_α_) distance. When this increases by ≈ 4 Å, approximately half a dozen water molecules enter the hydrophobic core, thereby stabilizing the open configuration for several ns. The simulations show that the lower extremity of the hydrophobic core, in close proximity to the Y26 side chain, is never completely devoid of water. It always retains 2–3 water molecules, which randomly make H-bonds with the hydroxyl group of Y26 and/or the carbonyl oxygen of G8. Our results suggest that the interaction of these water molecules with the Y26 hydroxyl group causes its aromatic side chain to fluctuate. We observed that these fluctuations randomly drive the aromatic side chain away from the core, thereby creating more space and opportunity for water to weaken and break the hydrophobic interactions and as a result allow additional water molecules to enter the core.

### Understanding the behavior of the hydrophobic core

The hydrophobic core in insulin is formed by the residues G8, L11, V12, L15, F24, Y26, I2_A_ and V3_A_ (see Fig 1*B*). While both the F24 and Y26 side chains contribute significantly to the formation of the hydrophobic core, they play very different roles in the opening process and it is important to understand the cause of this behavior. The F24 side chain is completely buried in the protein with no exposure to water and has a strong hydrophobic interaction with the L15 side chain. The position of F24 is further bolstered due to the residues flanking F24 forming H-bonds with the A-chain, i.e., G23(O)–N21_A_(N) and F25(N)–Y19_A_(O) (see Fig 1*C*). Thus the network of interactions around F24 result in a very stable hinge point at F24 and this explain why F24 retains its conformation during the opening of insulin, as observed in our MD simulations and also observed experimentally by Menting et al. [15]. In contrast, the side chain of Y26 is exposed to water and is more flexible due to the interaction of its hydroxyl group with the neighboring water molecules. Also T27 flanking Y26 is not involved in any stabilizing interactions with other residues. Thus the motion of Y26 is not restricted by its neighbor and the hydrophobic interactions between the Y26 side chain and the other residues in the core can be broken more easily, which triggers the opening of the BC-CT.

**Fig 8:**
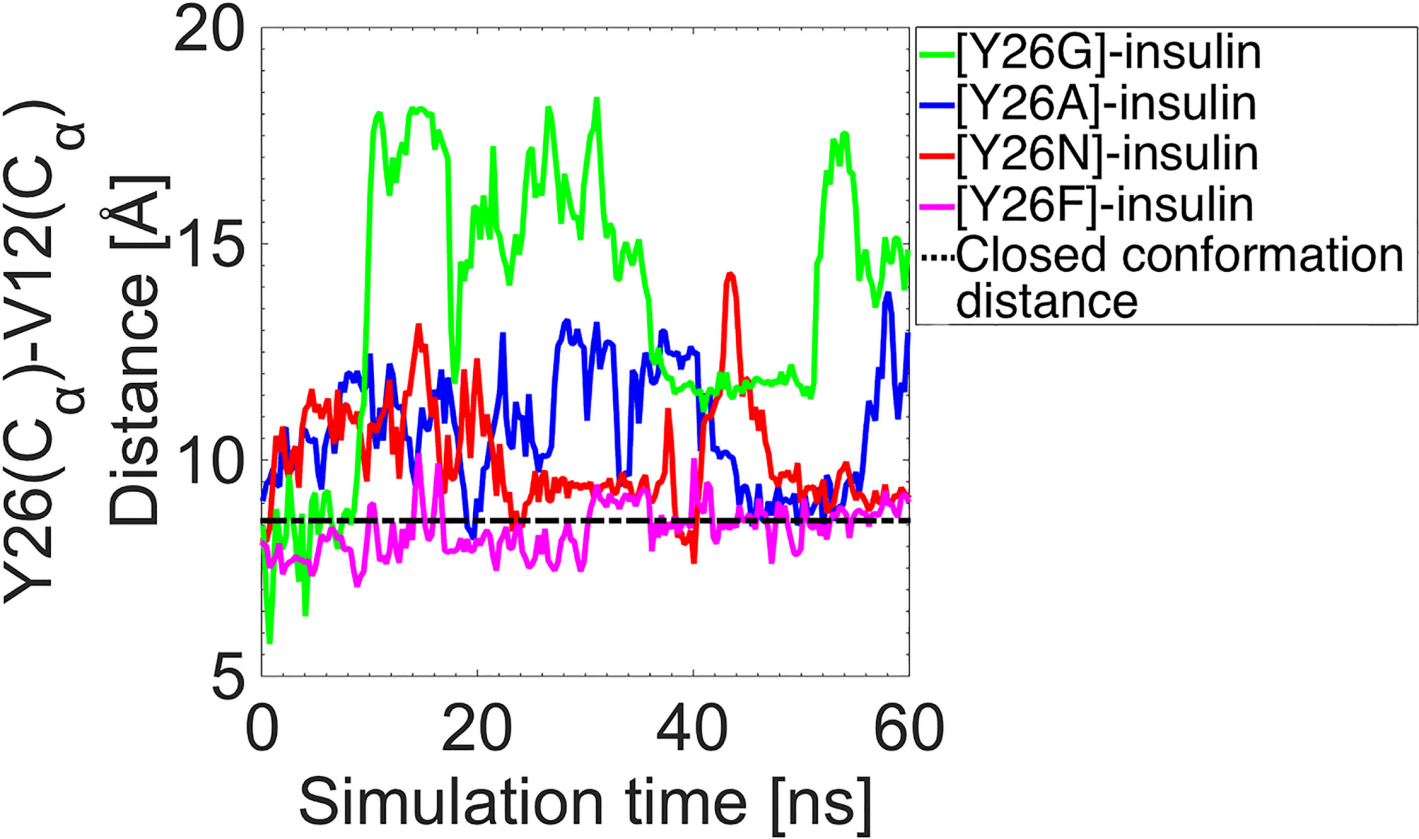
Effect of Y26 mutations on the BC-CT opening. The distance between the C_α_ atoms of the Y26–V12 residues, which determines the criterion for the BC-CT opening, for the Y26G, Y26A, Y26N and Y26F mutations. The closed conformation distance in WT insulin is shown for reference (black dashed line).

To examine the role of the Y26 side chain in the activation of insulin, we performed MD simulations of insulin with the mutations, Y26F, Y26N, Y26A and Y26G, for which there exists available mutagenesis data [18,22,24]. The effect of these mutations on the opening of the BC-CT is shown in Fig 8, while Table 3 lists the corresponding probability of each conformation. When the aromatic side chain of Y26 is replaced with a non-aromatic side chains, as in Y26N, Y26A and Y26G, the active form of insulin becomes the dominant conformation. In particular, the Y26G mutation causes the largest change from the WT insulin with the wide-open conformation becoming dominant (88% probability compared to 5%). The absence of an aromatic side chain weakens the hydrophobic interactions and thereby creates more space for water molecules to enter the hydrophobic core. The result in each case is a more flexible BC-CT and a much wider opening compared to that of WT insulin (cf. Fig 8). These simulation results corroborate the experimental results of Zakova et al. [24], where the mutations Y26G and Y26N were observed to result in an open conformation of insulin.

**Table 3:** Probabilities of the closed, open, and wide-open conformations of the BC-CT for WT and mutated insulin.

Insulin	Probability		
	Closed conformation	Open conformation	Wide-open conformation
WT	0.64	0.31	0.05
Y26F	0.66	0.34	0.00
Y26N	0.15	0.62	0.23
Y26G	0.07	0.05	0.88
Y26A	0.06	0.31	0.63

The role of the hydroxyl group in the Y26 side chain becomes evident upon closer inspection of the Y26F mutation. As seen from Fig 8 and Table 3, this mutation has negligible effect on the behavior of the opening, which is in agreement with the mutagenesis data [24]. Our simulation results show that the phenylalanine side chain occupies almost the same area as the tyrosine side chain and interacts similarly with the other residues of the core. A closer inspection of Fig 8 and Table 3 reveals a slightly more stable closed conformation for the Y26F mutation compared to WT insulin. We attribute this to the destabilizing effect of the hydroxyl-water interactions in WT insulin, which are absent in the Y26F mutation. Overall, our results indicate that Y26 is the critical residue in the activation of insulin. We note that the mutations that lead to a more active form of insulin have been found to generally also reduce its binding affinity to the IR [24], thus indicating that Y26 also plays an important role in binding of insulin. Of these mutations, only Y26N has been found to exhibit IR selectivity [24].

**Fig 9:**
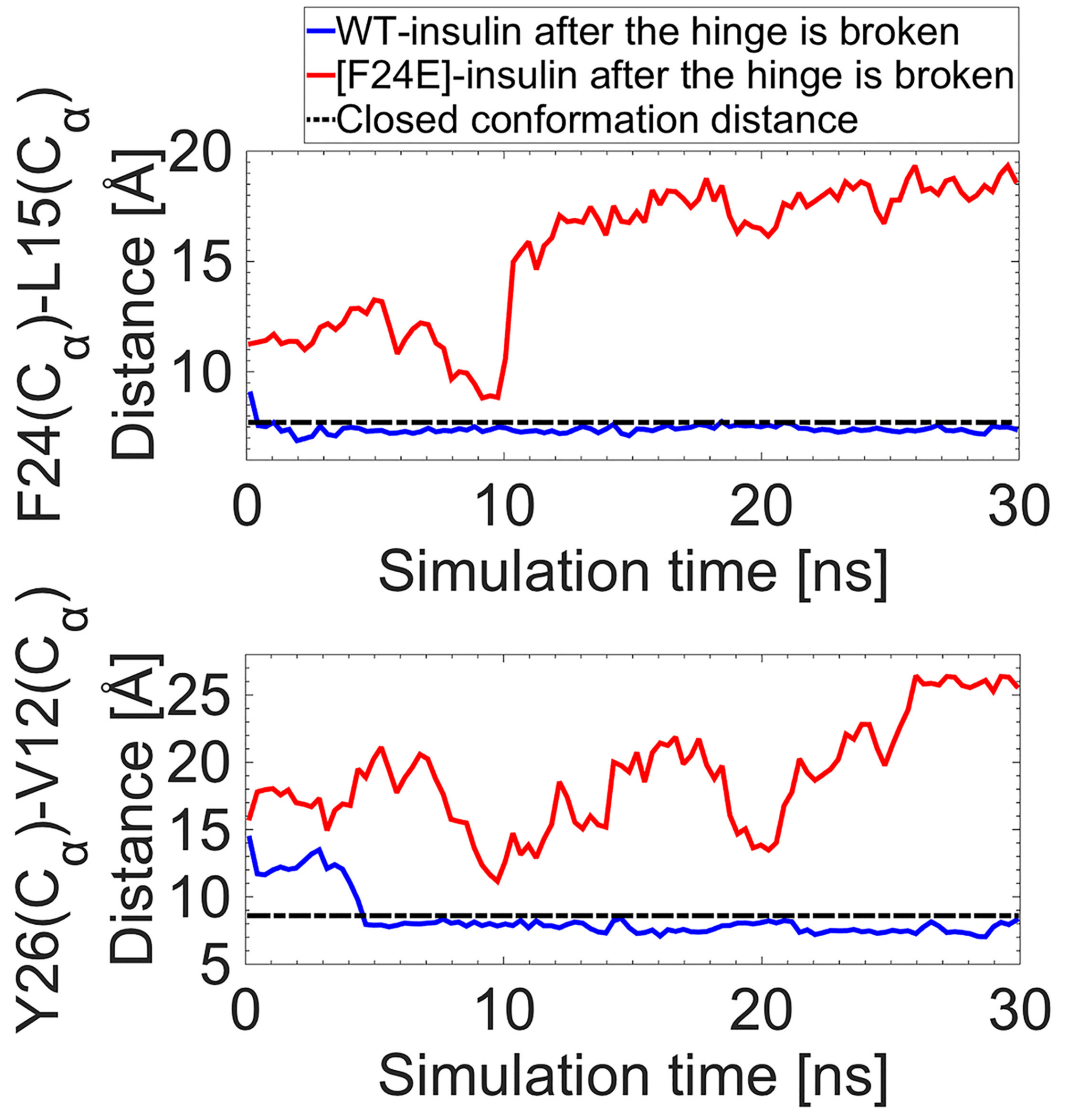
Stability of the hinge. Distances between the C_α_ atoms of the F24–L15 and Y26–V12 pairs, which represent the hinge and the BC-CT opening, respectively, for the WT (blue) and the mutated (red) insulin, after the hinge is broken and the protein is in its wide-open conformation. The trajectories are compared to the closed conformation distance of WT insulin (black dashed line).

We next investigate the stability of the hinge at the F24 residue. Initially, we performed 30 ns MD simulations of insulin with the mutations, F24A and F24H. In both cases, we found that the hinge is highly stable and did not break during the simulations. As a result, a more dynamic process was then used. A harmonic force is applied to the C_α_ atoms of the F24 and P28 residues for 200 ps to break the hinge and open the BC-CT in the WT insulin, followed by a 30 ns MD simulation of the protein with no forces applied. An identical simulation was performed for the F24E mutation to highlight the role of the aromatic side chain in stabilizing the hinge. Fig 9 shows the distances between the C_α_ atoms for the pairs F24–L15 and Y26–V12 for both the WT and the mutated insulin over the course of the simulation. The hinge, after breaking, is seen to reform in less than a nanosecond in WT insulin, which returns to its closed conformation after a few nanoseconds as indicated by the F24–L15 and Y26–V12 distances in Fig 9. Thus even after the hinge is broken via an external force, the F24 and Y26 aromatic side chains quickly return to their previous conformations, restoring the strong hydrophobic interactions with the other residues in the core and maintaining the insulin in its closed conformation for the rest of the simulation. In contrast, the F24E mutation disrupts the hydrophobic core by allowing water molecules to enter the core. This leads to the breakage of the hinge at F24 leaving insulin in a wide-open conformation during the whole simulation. We note that the F24E mutation results in a much wider opening of the BC-CT compared to the mutations of Y26, which clearly can be attributed to the breaking of the hinge.

## Conclusions

Our simulation results have revealed for the first time that the activation of insulin is stochastic in nature, with no obvious deterministic behavior. The BC-CT opens randomly and its dominant conformation is the closed/inactive one. The open conformation of the BC-CT does not match the receptor-bound interface. We have found an additional “wide-open” conformation, which uniquely enables insulin to bind to the IR. However, our simulations indicate that this wide-open conformation is a rare event (≈5% probability).

Our PMF results also reveal that the energy needed for the BC-CT residues to change their conformation from the closed to the open form is lowest for the residues near the C-terminal and increases towards the residues closer to the hinge. From this we deduce that the BC-CT opens like a zipper, i.e., the opening is initiated in the last residue and the other residues open progressively until the hinge is reached. We found that although the last residues of the BC-CT (P28–T30) are the most flexible, they are not capable of initiating the opening of insulin necessary for its activation. The hydrophobic interactions in the core maintain insulin in its closed conformation and the opening is feasible only when the core breaks at its outer limit (Y26). We also found that the opening process is triggered by the fluctuations in hydrophobic interactions, which allow entry of water molecules into the hydrophobic core, thereby disrupting the hydrophobic interactions between the nonpolar side chains of Y26 and the other residues in the core. The hinge at F24 was found to be very stable. We attribute this to bolstering of the hydrophobic interactions of F24 with the hydrogen bonds of the flanking residues.

Finally, our MD simulations have revealed that the Y26N, Y26A and Y26G mutations yield a higher probability of the wide-open conformation needed for IR binding, owing to more space being available in the core for water molecules to enter and weaken the hydrophobic interactions therein. Of these mutations, only the Y26N mutation was found to exhibit significant IR selectivity in previous experimental studies [24]. Thus, this particular mutation presents potential as a therapeutic insulin analogue. We plan to investigate this further with insulin-IR docking simulations.

In conclusion, our MD simulations provide new insights on the conformational dynamics of insulin that are necessary to enable its binding to the IR. In particular, we propose that binding of insulin to the IR most likely follows a two-stage process: the first is the stochastic opening of the BC-CT when insulin is in the vicinity of the αCT segment of the IR; in the second stage, conformational changes occur in both insulin (to the wide-open conformation) and the αCT segment of the IR, thereby facilitating switching of insulin from its free to bound conformation.
